# Association between rheumatoid factor and metabolic syndrome in general population

**DOI:** 10.1186/s13098-022-00914-w

**Published:** 2022-11-08

**Authors:** Lan Li, Donglai Feng, Jing Zeng, Peng Ye, Yao Chen, Dong Wei

**Affiliations:** 1 Medical Examination Center, The Second People’s Hospital of Chengdu, Chengdu, 610017 China; 2 Department of Endocrinology and Metabolism, Obesity and Metabolic Diseases Care Center, The Second People’s Hospital of Chengdu, Chengdu, 610017 China

**Keywords:** Metabolic syndrome, Rheumatoid factor, General population

## Abstract

**Background:**

Rheumatoid arthritis, metabolic syndrome (MS) and cardiovascular disease (CVD) are mutually connected. We aim to investigate the association between rheumatoid factor (RF) and MS in general population, explore the potential value of RF for assessment of metabolic status, and further provide a reference to the establishment of CVD primary prevention for this population.

**Methods:**

We assessed the health check-up subjects, accordance with the inclusive criteria, from 1 January 2015 to 31 October 2021 in a large refereed general hospital, in this retrospective study. Subjects were categorized into four groups according to their levels of RF. Multivariate logistic regression models along with the Odds ratio (OR) and Confidence interval (CI) values were used to measure the association between RF and MS.

**Results:**

A total of 13,690 subjects were analyzed. Prevalence of MS increased with RF level (P for trend < 0.001). Logistic regression analysis showed that, after adjusting for multiple covariates, RF level was significantly associated with MS prevalence (highest RF quartile: OR, 1.420; 95% CI 1.275,1.581, according to the revised National Cholesterol Education Program Adult Treatment Panel III criteria; OR, 2.355; 95% CI 2.085,2.660, according to the International Diabetes Federation criteria) (both P for trends < 0.001). Among the MS components, there were evidence of increasing trends for overweight/obesity (highest RF quartile: OR, 3.165; 95% CI 2.827,3.543) and hypertension (highest RF quartile: OR, 1.722; 95% CI 1.549,1.914) (both P for trends < 0.001), but decreasing trend for low high-density lipoprotein-cholesterol (highest RF quartile: OR, 0.245; 95% CI 0.214,0.281) (P for trend < 0.001), with increasing RF quartiles.

**Conclusions:**

RF level is associated with MS prevalence in general population. RF might be a valuable biomarker for assessment of metabolic status in this population. We should be aware of the cardiovascular risk for the higher-RF subjects.

**Supplementary Information:**

The online version contains supplementary material available at 10.1186/s13098-022-00914-w.

## Introduction

Rheumatoid arthritis (RA) is a systemic autoimmune disease characterized by excess morbidity and mortality from cardiovascular diseases (CVD). Moreover, the increased CVD risk seems like to be evident even in the subclinical stage [1–3]. Metabolic syndrome (MS) is defined as a set of manifestations that are contemplated as cardiovascular risk factors (obesity, hyperglycemia, dyslipidemia, and hypertension), that along with systemic chronic inflammation, contributes to CVD development. The prevalence of MS in RA has been widely investigated, and a higher prevalence of MS has been noted among RA patients [4, 5].

Rheumatoid factor (RF) is most commonly used as a part of the diagnostic criteria for RA. Besides, RF is also found in patients with other inflammatory conditions and is present in 1–5% of healthy subjects. In RA patients, both increased risk of CVD and prevalence of MS have been reported to be associated with the elevation of auto-antibodies and inflammatory markers, including RF [5–8].

In general population, RF has also been reported in few studies to be associated with cardiovascular morbidity and mortality [9–11]. However, the present results are still controversial. Until now, the association of RF and MS has never been discussed in general population. Thus, this study aims to investigate the relationship between RF and MS, explore the potential value of RF for assessment of metabolic status, and further provide a reference to establishment of CVD primary prevention for this population.

## Materials and methods

### Design and population

This retrospective cross-sectional study was conducted to investigate the association between serum RF levels and the presence of MS along with its components. The population of the present study consisted of men and women who had participated in a health-screening examination in the Second People’s Hospital of Chengdu, Sichuan province of China, between 1 January 2015 and 31 October 2021. The institutional review board of the hospital approved this study. The informed consent requirement was exempted because researchers only accessed retrospective database for analysis purposes. The reporting of this study conforms to the STROBE statement.

Inclusion criteria were men and women, 18 years old and above, tested for the serum level of RF, as well as all the components of MS. Exclusion criteria were arthritis, connective tissue diseases, liver diseases, any type of surgery and trauma during the prior month, malignant diseases, and acute infections. An additional file showed the flow diagram of participants selection (Additional file 1).

### Data collection and definitions

Study data included medical histories, physical examinations, and laboratory measurements. Medical and drug prescription histories were assessed by the examining physicians. Sitting blood pressure (BP), weight, and height were measured by trained nurses during the health examinations. BP was measured in the seated position after a period of rest with standard wrist electronic sphygmomanometers. Body mass index (BMI) was calculated as weight in kilograms divided by height in meters squared. Blood specimens were obtained from the antecubital vein after at least 8 h of fasting. Laboratory analyses were performed at the Laboratory Medicine Department of the Second People’s Hospital of Chengdu, Sichuan province of China. Serum levels of the following substances were measured: glucose, total cholesterol (TC), low-density lipoprotein-cholesterol (LDL-C), triglycerides (TG), high-density lipoprotein-cholesterol (HDL-C), aspartate aminotransferase (AST), alanine aminotransferase (ALT), gamma-glutamyl transpeptidase (GGT), high sensitivity-C reactive protein (hsCRP). The RF titer was measured by immunoturbidimetricassay (FUJIFILM Wako Pure Chemical Corporation), using a automatic biochemical analyzer (HITACHI LABOSPECT008AS). The normal range of RF were reported to be < 20 IU/ml.

We defined MS according to the International Diabetes Federation (IDF) criteria and the revised National Cholesterol Education Program (NCEP) Adult Treatment Panel (ATP) III criteria [12, 13]. According to the IDF criteria, MS was defined as central obesity, waist circumference (WC) ≥ 90 cm for Chinese men and ≥ 80 cm for Chinese women, along with two or more of the following abnormalities: (1) TG level of 150 mg/dL or receipt of specific treatment for this lipid abnormality; (2) HDL-C level of 40 mg/dL in men and 50 mg/dL in women or receipt of specific treatment for this lipid abnormality; (3) BP of 130/85 mmHg or receipt of treatment of previously diagnosed hypertension; and (4) Fasting blood glucose (FBG) level of 100 mg/dL or previously diagnosed type 2 diabetes. Owing to measurement limitation, WC were unavailable. We applied BMI (overweight/obesity) instead of WC (central obesity) and defined it according to Chinese Diabetes Society (CDS) criteria: BMI ≥ 25 kg/m^2^ [14]. The revised NCEP ATP III criteria classified a person with the MS in the same way as the IDF criteria, but did not require the presence of central obesity as an essential component.

### Statistical analysis

The results were expressed as mean ± (standard deviation, SD), median (interquartile range, IQR), or number (percentage). Categories of serum RF comprised the following quartiles: ≤ 1.90 (Q1 group), > 1.90 and ≤ 2.90 (Q2 group), > 2.90 and ≤ 5.90 (Q3 group), and > 5.90 (Q4 group) IU/ml. Comparison of categorical variables was performed using χ^2^ test. Differences among groups were compared using one-way analysis of variance. Comparisons among different groups were performed using χ^2^ Mantel–Haenszel test for trend in the distribution of MS and its components. Univariate and multivariate logistic regression analyses were used to determine the association between the presence of MS and RF level. Variables were included into multivariate analysis because of their known clinical importance for MS or RF. Models were presented as unadjusted (model 1), minimally adjusted (model 2 adjusted for age, sex) and multivariate adjusted (model 3 adjusted for age, sex, AST, ALT, GGT). Subsequently, hsCRP were further adjusted (model 4) to explore the relevant mechanism of the potential association. All reported probability values were 2-tailed, and a P value < 0.05 was considered statistically significant. SPSS version 22.0 (IBM Corp.) were used for calculations.

## Results

### Population characteristics by RF level

A total of 16,054 health-screening subjects were included. Among this population, 2364 were excluded, and the other 13,690 were included in the final analysis. The median hsCRP and RF values of the whole study population were 1.00 (IQR, 0.90–2.00) and 2.90 (IQR, 1.90–5.90). The mean age of the population was 47.43 ± 11.78 years. The prevalence of MS were 26.57% according to the IDF and 41.52% according to the revised NCEP ATP III criteria. Subjects with higher RF quartiles had a higher BMI (P for trend < 0.001). The levels of BP, FBG, HDL-C and TG showed the trends of increasing in subjects with higher RF quartiles (all P for trends < 0.01) (Table 1). The prevalence of MS according to both of the revised NCEP ATP III (33.57% in Q1, 38.12% in Q2, 45.88% in Q3, and 48.51% in Q4; P for trend < 0.001) and IDF (17.62% in Q1, 22.70% in Q2, 26.12% in Q3, and 39.86% in Q4; P for trend < 0.001) criteria were higher in subjects with higher RF level. Table 1 showed the demographics and clinical characteristics of this study population across quartiles of RF level.

**Table 1:** Demographics and clinical characteristics of the study population by rheumatoid factor level

Variables	Total	Q1	Q2	Q3	Q4	P for trend
Age (years)	47.43 ± 11.78	42.12 ± 11.66	47.94 ± 8.99	46.77 ± 10.33	52.87 ± 13.15	< 0.001
Gender (male%)	6697 (48.92%)	1704 (49.78%)	1584 (46.28%)	1676 (48.98%)	1733 (50.64%)	0.166
BMI (kg/m^2^)	24.28 ± 2.95	23.21 ± 2.31	23.97 ± 2.77	24.79 ± 2.86	25.02 ± 3.38	< 0.001
SP (mmHg)	130.79 ± 17.87	127.45 ± 14.77	130.40 ± 18.59	133.28 ± 19.96	132.02 ± 17.23	< 0.001
DP (mmHg)	76.96 ± 12.29	76.31 ± 10.42	74.73 ± 11.55	78.00 ± 13.40	78.77 ± 13.13	< 0.001
LDL-C (mg/dL)	114.74 ± 30.36	114.07 ± 30.70	118.17 ± 31.51	113.64 ± 30.63	113.06 ± 28.27	0.001
TG ( mg/dL)	154.77 ± 115.43	141.75 ± 96.81	142.97 ± 75.84	167.88 ± 146.84	166.20 ± 126.23	< 0.001
TC (mg/dL)	194.00 ± 35.30	193.62 ± 34.42	197.35 ± 35.47	193.42 ± 38.51	191.62 ± 32.27	< 0.001
HDL-C (mg/dL)	52.82 ± 9.33	52.11 ± 10.23	53.47 ± 10.20	52.66 ± 8.33	53.02 ± 8.33	0.007
FBG (mg/dL)	103.87 ± 29.98	97.29 ± 23.66	107.76 ± 41.30	104.60 ± 22.62	105.85 ± 27.50	< 0.001
AST (U/L)	20.89 ± 15.96	17.17 ± 7.67	16.30 ± 8.61	26.03 ± 21.81	24.08 ± 18.40	< 0.001
ALT (U/L)	20.62 ± 17.04	16.80 ± 6.83	16.01 ± 8.17	25.54 ± 23.68	24.15 ± 20.36	< 0.001
GGT (U/L)	24.33 ± 16.83	20.02 ± 7.11	19.36 ± 8.13	29.36 ± 23.26	28.55 ± 19.72	< 0.001
hsCRP (mg/L)	1.00 (0.90–2.00)	1.00 (0.90–2.00)	1.00 (1.00–2.00)	1.00 (0.90–2.55)	1.70 (1.00–4.20)	< 0.001
Overweight/obesity (%)	5106 (37.30%)	768 (22.44%)	1207 (35.26%)	1439 (42.05%)	1692 (49.44%)	< 0.001
Hypertension (%)	7030 (51.35%)	1377 (40.22%)	1668 (48.73%)	2039 (59.59%)	1946 (56.87%)	< 0.001
Hypertriglyceridaemia (%)	8658 (63.24%)	2244 (65.56%)	2151 (62.84%)	2224 (65.00%)	2039 (59.59%)	< 0.001
Low HDL-C (%)	3034 (22.16%)	1085 (31.70%)	608 (17.76%)	799 (23.35%)	542 (15.84%)	< 0.001
Hyperglycaemia (%)	5328 (38.92%)	1150 (33.60%)	1370 (40.02%)	1378 (40.27%)	1430 (41.79%)	< 0.001
MS (%)						
Revised NCEP ATP III criteria	5684 (41.52%)	1149 (33.57%)	1305 (38.12%)	1570 (45.88%)	1660 (48.51%)	< 0.001
IDF criteria	3638 (26.57%)	603 (17.62%)	777 (22.70%)	894 (26.12%)	1364 (39.86%)	< 0.001

BMI, body mass index; SP, systolic pressure; DP, diastolic pressure; LDL-C, low-density lipoprotein-cholesterol; TG, triglycerides; TC, total cholesterol; HDL-C, high-density lipoprotein-cholesterol; FBG, fasting blood glucose; AST, aspartate aminotransferase; ALT, alanine aminotransferase; GGT, gamma-glutamyl transpeptidase; hsCRP, high sensitivity-C reactive protein; RF, rheumatoid factor; MS, metabolic syndrome; NCEP, National Cholesterol Education Program; ATP, Adult Treatment Panel; IDF, International Diabetes Federation

### Association between MS, according to the revised NCEP ATP III and IDF criteria, and RF

In the unadjusted model, ORs for MS in Q4 were 1.865 (95% CI 1.691,2.056) according to the revised NCEP ATP III and 3.100 (95% CI 2.773,3.465) according to the IDF (both P for trends < 0.001) criteria compared with Q1. After adjusting for age and sex (model 2), the association (Q4: OR,1.825; 95% CI 1.647,2.023, according to the revised NCEP ATP III criteria; OR, 2.697; 95% CI 2.397,3.035, according to the IDF criteria) remained significant (both P for trends < 0.001). After additionally adjusting for AST, ALT and GGT (model 3), the association were still significant (Q4: OR, 1.420; 95% CI 1.275,1.581, according to the revised NCEP ATP III criteria; OR, 2.355; 95% CI 2.085,2.660, according to the IDF criteria) (both P for trends < 0.001). In model4 ( further adjusting for hsCRP), multivariate analysis also showed significant association (Q4: OR, 1.175; 95% CI 1.052,1.313, according to the revised NCEP ATP III criteria; OR, 1.776; 95% CI 1.566,2.015, according to the IDF criteria) (P for trend < 0.001 according to the IDF criteria; P for trend = 0.002 according to the revised NCEP ATP III criteria) (Table 2).

**Table 2:** Odd ratios for the prevalence of metabolic syndrome according to rheumatoid factor quartiles

			Q2	Q3	Q4	P for trend
The revised NCEP ATP III criteria	^Model 1^	^OR^	1.219 (1.105,1.346)	1.678 (1.522,1.850)	1.865 (1.691,2.056)	< 0.001
		P	< 0.001	< 0.001	< 0.001	
	^Model 2^	OR	1.211 (1.095,1.339)	1.661 (1.505,1.834)	1.825 (1.647,2.023)	< 0.001
		P	< 0.001	< 0.001	< 0.001	
	^Model 3^	OR	1.268 (1.144,1.405)	1.308 (1.180,1.450)	1.420 (1.275,1.581)	< 0.001
		P	< 0.001	< 0.001	< 0.001	
	^Model 4^	OR	1.197 (1.077,1.329)	1.135 (1.021,1.261)	1.175 (1.052,1.313)	0.002
		P	0.001	0.019	0.004	
The IDF criteria	^Model 1^	OR	1.373 (1.219,1.547)	1.654 (1.472,1.858)	3.100 (2.773,3.465)	< 0.001
		P	< 0.001	< 0.001	< 0.001	
	^Model 2^	OR	1.312 (1.162,1.482)	1.587 (1.410,1.787)	2.697 (2.397,3.035)	< 0.001
		P	< 0.001	< 0.001	< 0.001	
	^Model 3^	OR	1.406 (1.242,1.590)	1.437 (1.272,1.624)	2.355 (2.085,2.660)	< 0.001
		P	< 0.001	< 0.001	< 0.001	
	^Model 4^	OR	1.291 (1.137,1.466)	1.151 (1.014,1.306)	1.776 (1.566,2.015)	< 0.001
		P	< 0.001	0.029	< 0.001	

Model 1: unadjusted; Model 2: adjusted for age and sex; Model 3: adjusted for age, sex, aspartate aminotransferase, alanine aminotransferase, and gamma-glutamyl transpeptidase; Model 4: adjusted for age, sex, aspartate aminotransferase, alanine aminotransferase, gamma-glutamyl transpeptidase and high sensitivity-C reactive protein

NCEP, National Cholesterol Education Program; ATP, Adult Treatment Panel; IDF, International Diabetes Federation

### Association between the components of MS and RF

Only results from model 3 (adjusted for age, sex, AST, ALT and GGT) were described here (Table 3). In multivariate analysis, there were evidence of increasing trends for hypertension (Q4: OR, 1.722; 95% CI 1.549,1.914) (P for trend < 0.001) and overweight/obesity (Q4: OR, 3.165; 95% CI 2.827,3.543) (P for trend < 0.001), but decreasing trend for low HDL-C (Q4: OR, 0.245; 95% CI 0.214,0.281) (P for trend < 0.001), with increasing RF quartiles. There were no significant and mild (but significant) differences in the prevalence of hyperglycaemia (P for trend = 0. 910) and hypertriglyceridaemia (P for trend = 0.001) respectively, across quartiles of RF.

**Table 3:** Odd ratios for the prevalence of metabolic syndrome components according to rheumatoid factor quartiles

			Overweight/Obesity	Hypertension	Hyperglycaemia	Low HDL-C	Hypertriglyceridaemia
Q2		OR	1.939 (1.739,2.162)	1.486 (1.345,1.642)	1.403 (1.266,1.555)	0.337 (0.298,0.382)	1.004 (0.906,1.112)
	P		< 0.001	< 0.001	< 0.001	< 0.001	0.944
Q3		OR	2.552 (2.287,2.848)	1.829 (1.651,2.025)	1.007 (0.907,1.118)	0.500 (0.442,0.564)	0.968 (0.871,1.075)
	P		< 0.001	< 0.001	0.897	< 0.001	0.539
Q4		OR	3.165 (2.827,3.543)	1.722 (1.549,1.914)	1.135 (1.018,1.266)	0.245 (0.214,0.281)	0.847 (0.760,0.943)
	P		< 0.001	< 0.001	0.022	< 0.001	0.002
	P for trend		< 0.001	< 0.001	0.910	< 0.001	0.001

Model was adjusted for age, sex, adjusted for age, sex, aspartate aminotransferase, alanine aminotransferase, and gamma-glutamyl transpeptidase

HDL-C, high-density lipoprotein-cholesterol

## Discussion

It is unknown whether elevated levels of RF in the general population are associated with increased prevalence of MS. Herein, we demonstrate that the higher RF is associated with a greater MS prevalence in 13,690 Chinese who participated in a health-screening examination. The ORs for MS increase with the RF levels (about 1.42 according to the revised NCEP ATP III and 2.36 according to the IDF criteria, in the highest RF quartile compared to the lowest RF quartile).

The underlying causal mechanism of the association between RF and MS remains unclear. The possible functions of RF include the activation of the chronic inflammatory pathway. In fact, many conditions involving in chronic inflammation are associated with RF elevation [15]. In our study, ORs for MS, especially according to the IDF criteria, in higher RF quartiles markedly decreased after hsCRP level had been adjusted for. Thus, it is likely that elevated RF indicates low-grade chronic inflammation caused by immune complexes, which plays an important part. Nevertheless, we still notice that the association between RF and MS were significant after adjusting for hsCRP. This suggests that other biological pathways independent of inflammation are involved in, such as altered humoral immunity. In fact, mechanism of the association between RF and MS might be more complex, because adiposity in MS subjects wound simultaneously trigger the release of adipokines or other mediators and influence the production of auto-immune antibodies [3, 4].

As we known, none has discussed the association between RF and MS in general population this before. Few studies have addressed the association between RF and CVD morbidity and mortality in the general population. In the study by Tomasson et al. [10], RF was associated with greater all-cause and cardiovascular mortality in the general population, although the study included subjects with inflammatory arthritis. Edwards et al. [16] had reported that higher RF level was an independent risk factor for coronary artery disease (CAD) in men from the general population. Nevertheless, Nossent et al. [11] had reported that RF detected by Latex agglutination did not independently predict CVD or death in the general population in the Busselton Health Survey.

Data on the association between RF and the metabolic components are mostly from the secondary or baseline analysis of cohort studies, and are still conflicting. In the study by Tomasson et al. [10], as discussed above, RF-positive subjects had greater diabetes prevalence and lower cholesterol levels than RF-negative subjects in a general population cohort. In the cohort study to determine whether RF was associated with mortality from all-cause, CVD and cancer in Korean healthy adults, obesity and TG and HDL-C levels were not significantly different between RF-positive and RF-negative subjects at baseline [9]. Jafarzadeh et al. [17] conducted the cross-sectional study aiming to evaluate the serum levels of several autoantibodies in patients with ischemic heart disease (IHD) compared to healthy population and found no difference in RF levels in IHD patients with traditional risk factors, including hypertension, dyslipidemia, diabetes, and those without a certain risk factor. In the present analysis, overweight/obesity was most strongly associated with RF, which, along with insulin resistance, has been widely considered as the key point of MS. This suggests the important role of overweight/obesity in connecting the elevated RF with the greater MS prevalence, and also explain the stronger association between RF and MS by the IDF, which takes obesity as a essential component, than MS by the revised NCEP ATP III. Surprisingly, low HDL-C and hypertriglyceridaemia were negatively correlated with RF here. It had been reported that, HDL-C levels were persistently low but, along with its atheroprotective roles, fluctuate as a consequence of alterations in disease activity in RA. TG levels in RA were still controversial. Small study sample size and differences in the populations had been explained for this controversy [18, 19]. But here, mechanism of the association between relatively higher RF and higher HDL-C and lower TG in general population needs being further explored. In addition, no significant association of hyperglycaemia (after adjusting for covariates)were obtained. There might be more influential factors for blood glucose.

To our knowledge, this is the first study exploring the association between RF and MS in Chinese general population, who have a relatively healthy status. And the results are persuasive with the large study sample size and the sufficient statistical power. However, our study has several limitations. Firstly, we could not completely exclude all patients with RA as well as other diseases likely to influence the results by the way of self-statement. Moreover, individual history of smoking and drinking were not collected. Thus, this might result in some degree of residual confounding. Secondly, we use BMI instead of WC to define MS according to CDS criteria. This might not accurately reflect the level of visceral fat as well as the status of metabolism. Thirdly, this is an retrospective cross-sectional analysis, which could not declare the casual function of RF on the incidence of MS and its influence on CVD risk. In the further, prospective and mechanism studies need to be carried out.

## Conclusions

RF level is associated with MS prevalence in general population. RF might be a valuable biomarker for assessment of metabolic status in this population. On the other hand, we should explain the meaning of RF in those with MS carefully. This study helps to provide a reference to the establishment of CVD primary prevention for this population.

## Supplementary Information

**Additional file 1. **Flow diagram of participants selection.

## Data Availability

The datasets generated during this study are available from the correspondences on reasonable request.

## Abbreviations

ALT: Alanine aminotransferase
AST: Aspartate aminotransferase
ATP: Adult treatment panel
BP: Blood pressure
BMI: Body mass index
CAD: Coronary artery disease
CDS: Chinese Diabetes Society
CI: Confidence interval
CVD: Cardiovascular disease
FBG: Fasting blood glucose
GGT: Gamma-glutamyl transpeptidase
HDL-C: High-density lipoprotein-cholesterol
hsCRP: High sensitivity-C reactive protein
IDF: International Diabetes Federation
IHD: Ischemic heart disease
IQR: Interquartile range
LDL-C: Low-density lipoprotein-cholesterol
MS: Metabolic syndrome
NCEP: National Cholesterol Education Program
OR: Odds ratio
RA: Rheumatoid arthritis
RF: Rheumatoid factor
SD: Standard deviation
TC: Total cholesterol
TG: Triglycerides
WC: Waist circumference
