# Supplementary figures and images for: Association between rheumatoid factor and metabolic syndrome in general population
Source: Diabetol Metab Syndr. 2022 Nov 8;14:165. doi: 10.1186/s13098-022-00914-w (PMC9641854; doi:10.1186/s13098-022-00914-w)

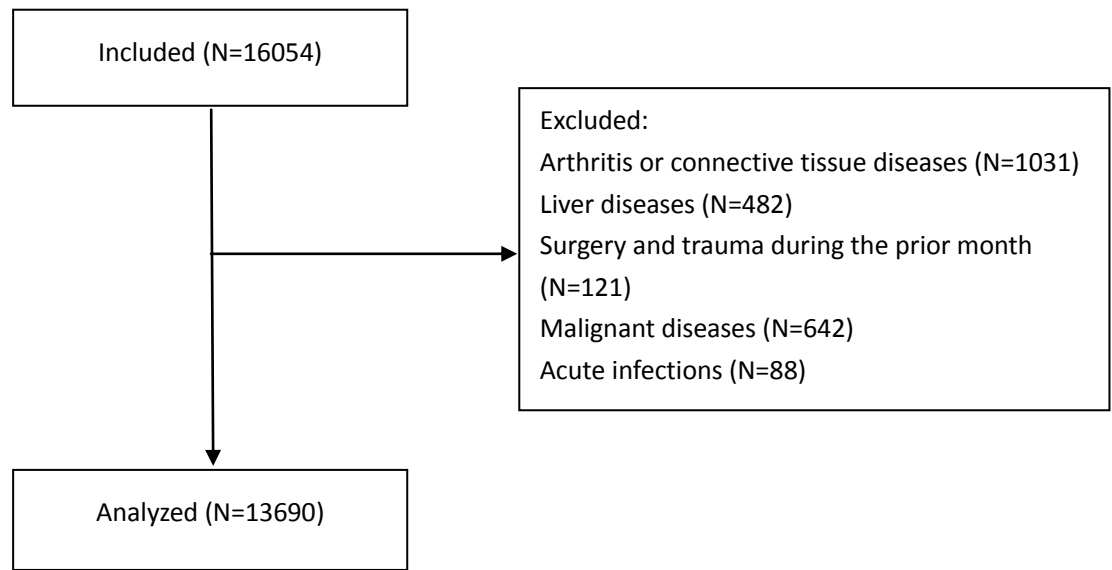

Flow diagram of participants selection

Supplement: Supplementary file 1 — Additional file 1. Flow diagram of participants selection. [file 13098_2022_914_MOESM1_ESM.pdf]
